# Development and Validation of Nomogram Models for Postoperative Pneumonia in Adult Patients Undergoing Elective Cardiac Surgery

**DOI:** 10.3389/fcvm.2021.750828

**Published:** 2021-10-11

**Authors:** Dashuai Wang, Xing Chen, Jia Wu, Sheng Le, Fei Xie, Ximei Li, Hongfei Wang, Xiaofan Huang, Anchen Zhang, Xinling Du

**Affiliations:** ^1^Department of Cardiovascular Surgery, Union Hospital, Tongji Medical College, Huazhong University of Science and Technology, Wuhan, China; ^2^Key Laboratory for Molecular Diagnosis of Hubei Province, The Central Hospital of Wuhan, Tongji Medical College, Huazhong University of Science and Technology, Wuhan, China; ^3^Department of Cardiovascular Surgery, First Affiliated Hospital of Zhengzhou University, Zhengzhou, China; ^4^Department of Nursing, Huaihe Hospital of Henan University, Kaifeng, China; ^5^Department of Cardiology, The Central Hospital of Wuhan, Tongji Medical College, Huazhong University of Science and Technology, Wuhan, China

**Keywords:** pneumonia, risk factor, cardiac surgery, nomogram, prediction model

## Abstract

**Background:** Postoperative pneumonia (POP) is a frequent complication following cardiac surgery, related to increased morbidity, mortality and healthcare costs. The objectives of this study were to investigate the risk factors associated with POP in adults undergoing elective cardiac surgery and to develop and validate nomogram models.

**Methods:** We conducted a multicenter retrospective study in four cardiac centers in China. Adults operated with elective open-heart surgery from 2016 to 2020 were included. Patients were randomly allocated to training and validation sets by 7:3 ratio. Demographics, comorbidities, laboratory data, surgical factors, and postoperative outcomes were collected and analyzed. Risk factors for POP were identified by univariate and multivariate analysis. Nomograms were constructed based on the multivariate logistic regression models and were evaluated with calibration, discrimination and decision curve analysis.

**Results:** A total of 13,380 patients meeting the criteria were included and POP developed in 882 patients (6.6%). The mortality was 2.0%, but it increased significantly in patients with POP (25.1 vs. 0.4%, *P* < 0.001). Using preoperative and intraoperative variables, we constructed a full nomogram model based on ten independent risk factors and a preoperative nomogram model based on eight preoperative factors. Both nomograms demonstrated good calibration, discrimination, and were well validated. The decision curves indicated significant clinical usefulness. Finally, four risk intervals were defined for better clinical application.

**Conclusions:** We developed and validated two nomogram models for POP following elective cardiac surgery using preoperative and intraoperative factors, which may be helpful for individualized risk evaluation and prevention decisions.

## Introduction

Postoperative pneumonia (POP) is a prevalent infection in patients undergoing elective cardiac surgery (ECS), strongly related to higher rates of morbidity and mortality (1, 2). As a result, healthcare costs and resource utilization are also evidently increased (3). The prevalence rate of POP after cardiac surgery varied significantly in previous reports and the true incidence may be underestimated (4, 5).

Globally, many studies have been carried out to explore risk factors for POP following cardiac surgery due to its high prevalence and poor outcome (5–8). Some significant predictors have been identified and several risk prediction models have been established (6–9). However, the baseline characteristics and comorbidities of patients have changed greatly these years due to great advances in surgical and anesthesia techniques (10). Numerous studies were nearly decades old and some of the earlier drawn conclusions may now be obsolete due to the narrow patient selection and small samples. New large-scale persuasive researches on this topic have been uncommon in recent years. To our knowledge, most published studies were conducted in coronary artery bypass grafting or mixed surgeries, but none were designed specific for patients undergoing ECS. It's still in urgent need to establish a convincing prediction model to predict the probability of POP after ECS, and it may make more sense than previous prediction models as the vast majority of cardiac surgery are elective procedures.

The objectives of this study were to identify independent risk factors for the occurrence of POP in adult patients who underwent ECS and to develop and validate two nomogram models to facilitate individualized risk assessment and reasonable prevention.

## Materials and Methods

### Study Population

This is a multicenter, observational, retrospective study. Adult patients who underwent elective open-heart surgery from 2016 to 2020 in four tertiary care centers in China were included. Patients with one or more of the listed conditions were excluded: (1) emergent cardiac surgery; (2) acquired pneumonia within 2 weeks before surgery; (3) discharged or died within postoperative 48 h; and (4) immunosuppression, immune deficiency, or organ transplantation.

### Data Collection

We collected clinical data using the hospital's electronic medical record systems. Demographics, comorbidities, laboratory values, and intraoperative variables were collected and analysed. Postoperative variables were also collected and compared between groups.

### Definition of Important Variables

POP was diagnosed based on the recommendations of the 2016 clinical practice guideline (11). Smoking history referred to previous daily or current smoking. Chronic obstructive pulmonary disease (COPD) referred to FEV1/FVC ≤0.7. Drinking history referred to drinking once or more a week more than 1 year, current drinking, or quitting within 3 years. Diabetes mellitus referred to previous diagnosis, use of diabetic medication, fasting glucose ≥7.0 mmol/L, or random glucose ≥11.1 mmol/L. Hypertension referred to previous diagnosis, using antihypertensive medication, or blood pressure ≥140/90 mmHg. Renal insufficiency referred to previous diagnosis or serum creatinine level >110 μmol/L. Body mass index was defined as the ratio of weight to height squared.

### Statistical Analysis

Statistical analyses were performed using SPSS (IBM SPSS Statistics, version 26) and R software (version 4.0.4, https://www.R-project.org/). It was considered statistically significant when two-tailed *P*-value < 0.05.

The overall dataset was randomly allocated to the training set and the validation set at a ratio of 7:3. The training set was used to develop the model, whereas the validation set was used to validate the model. Continuous variables in normal distribution were presented as means ± standard deviations, and those that were skewed were presented as medians with inter-quartile ranges. Categorical variables were presented as counts with percentages. Missing data were handled using multiple imputation. We first conducted univariate analysis in the training set to screen potential risk factors. Continuous variables with homogeneous variance and normal distribution were compared by Student's *t*-test, otherwise, the Mann-Whitney U-test was used. Categorical variables were compared using the chi-square test or Fisher's exact test. Factors screened were further applied for a forward stepwise multivariate logistic regression analysis to identify independent predictors. A nomogram was then constructed to assess the risk of POP after ECS based on the multivariate model.

Both Hosmer-Lemeshow goodness-of-fit test and visual inspection were used to assess the calibration of the model. The area under the receiver operating characteristic (ROC) curve (AUC) were used to evaluate the discrimination. Decision curve analysis was used to evaluate the clinical utility. The internal validation was performed using 1,000 bootstrap replicates. The external validation was performed in the validation set.

## Results

### Demographic Characteristics

**Figure 1:**
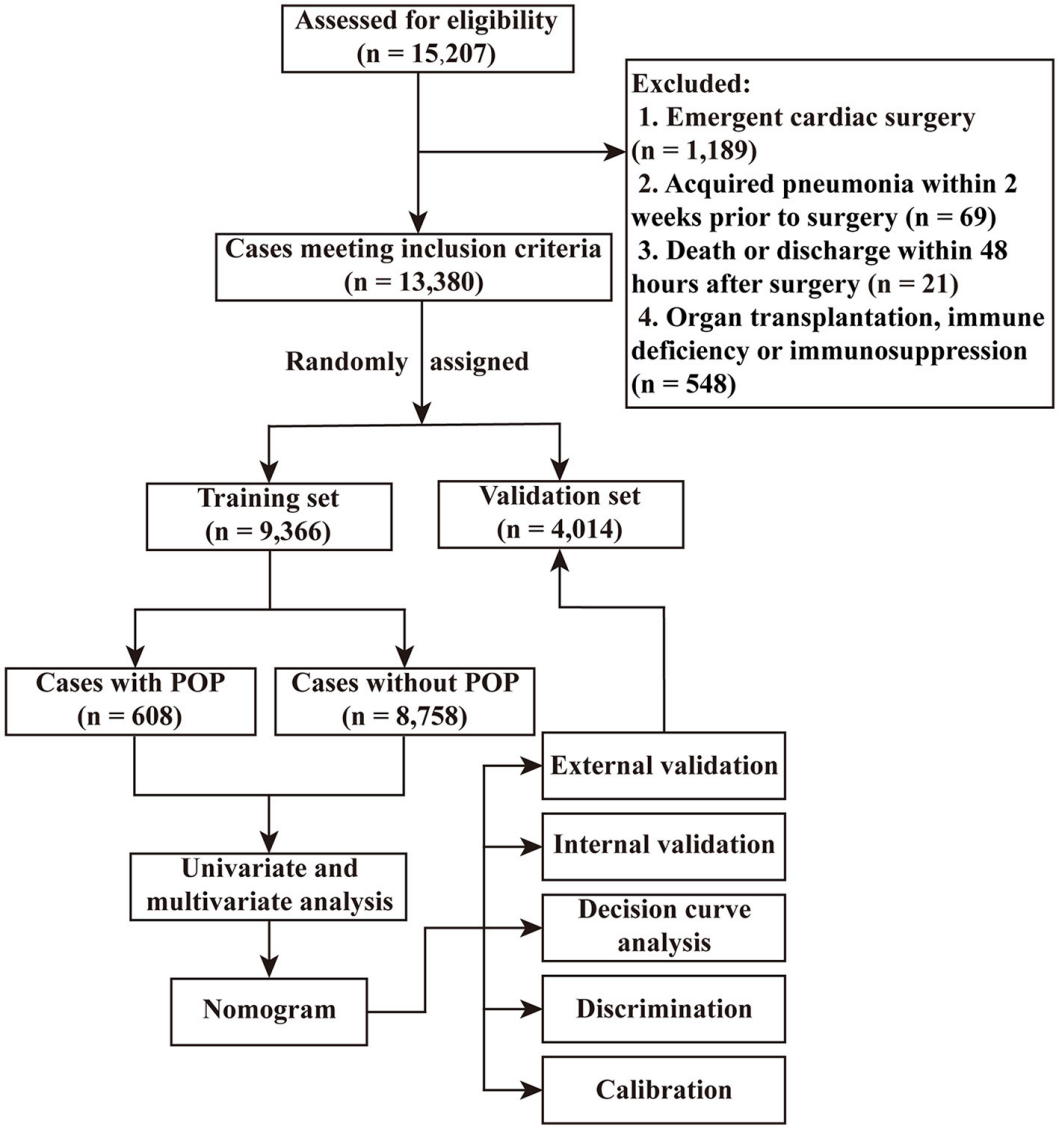
Flow chart of the study. POP, postoperative pneumonia.

Among the 15,207 adults undergoing cardiac surgery, 1,189 patients experienced emergency surgery, 69 patients had pneumonia within 2 weeks before surgery, 21 patients died or discharged within 48 h after surgery, and 548 patients experienced organ transplantation, immunosuppression, or immune deficiency. The remaining 13,380 cases meeting the inclusion criteria were further analyzed (Figure 1), including 6,122 cases from Union Hospital of Tongji Medical College of Huazhong University of Science and Technology, 5,735 cases from First Affiliated Hospital of Zhengzhou University, 1,256 patients from The Central Hospital of Wuhan, and 267 cases from Huaihe Hospital of Henan University. The mean age of these patients was 51.55 ± 13.16 years, in which 54.0% were males. The incidence rate of POP after ECS was 6.6%.

The study population had different patient details and comorbidities. Patients with smoking history accounted for 27.1%, drinking history 20.2%, COPD 11.5%, hypertension 25.0%, diabetes mellitus 7.9%, renal insufficiency 7.3%, gastrointestinal tract disease 8.1%, atrial fibrillation 19.4%, cardiac surgery history 6.9%. The average CPB time was 100 (77, 129) min and the average aortic cross clamp time was 67 (47, 88) min. Intraoperative RBC transfusions were used in 71.8% of the patients and the average volume was 1.0 (0, 3.0) units. The characteristics, comorbidities and surgical factors of the patients were similar in the training and validation datasets (Table 1). The POP incidence was respectively, 6.5 and 6.8% in the two sets (*P* = 0.48).

**Table 1:** Comparison of characteristics between the training and validation sets.

**Characteristic**	**All cases**	**Training set**	**Validation set**	***P*** **-value**
	***n*** **= 13,380 (%)**	***n*** **= 9,366 (%)**	***n*** **= 4,014 (%)**	
**Demographics**				
Age (years)	51.55 ± 13.16	51.57 ± 13.21	51.49 ± 13.04	0.762
Male	7,225 (54.0)	5,094 (54.4)	2,131 (53.1)	0.167
Body mass index (kg/m^2^)	23.13 ± 3.29	23.14 ± 3.30	23.10 ± 3.29	0.485
Smoking history	3,627 (27.1)	2,552 (27.2)	1,075 (26.8)	0.578
Drinking history	2,705 (20.2)	1,888 (20.2)	817 (20.4)	0.796
**Underlying conditions**				
Hypertension	3,345 (25.0)	2,338 (25.0)	1,007 (25.1)	0.879
Diabetes mellitus	1,059 (7.9)	714 (7.6)	345 (8.6)	0.056
Chronic obstructive pulmonary disease	1,533 (11.5)	1,055 (11.3)	478 (11.9)	0.284
Cerebrovascular disease	2,337 (17.5)	1,634 (17.4)	703 (17.5)	0.925
Peripheral vascular disease	2,931 (21.9)	2,048 (21.9)	883 (22.0)	0.866
Renal insufficiency	971 (7.3)	681 (7.3)	290 (7.2)	0.925
Gastrointestinal tract disease	1,088 (8.1)	768 (8.2)	320 (8.0)	0.659
Atrial fibrillation	2,591 (19.4)	1,802 (19.2)	789 (19.7)	0.576
General surgery history	3,896 (29.1)	2,753 (29.4)	1,143 (28.5)	0.284
Cardiac surgery history	924 (6.9)	640 (6.8)	284 (7.1)	0.613
New York Heart Association class III-IV	2,274 (17.0)	1,566 (16.7)	708 (17.6)	0.195
Pulmonary artery hypertension	3,905 (29.2)	2,716 (29.0)	1,189 (29.6)	0.468
Pericardial effusion	1,747 (13.1)	1,203 (12.8)	544 (13.6)	0.265
Left ventricular ejection fraction (%)	62 (57, 67)	62 (57, 67)	62 (57, 67)	0.356
**Laboratory values**				
White blood cell count (× 10^9^/L)	5.67 (4.72, 6.80)	5.66 (4.72, 6.80)	5.68 (4.74, 6.77)	0.685
Red blood cell count (× 10^12^/L)	4.30 (3.94, 4.66)	4.30 (3.94, 4.66)	4.29 (3.93, 4.65)	0.095
Hemoglobin (g/l)	130 (118, 141)	130 (118, 141)	129 (118, 141)	0.478
Serum creatinine (μmol/L)	71.2 (60.5, 83.9)	71.2 (60.5, 84.0)	71.3 (60.4, 83.3)	0.669
Serum albumin (g/L)	40.58 ± 3.81	40.58 ± 3.80	40.60 ± 3.83	0.769
Serum globulin (g/L)	24.64 ± 4.38	24.65 ± 4.38	24.62 ± 4.36	0.748
**Operative variables**				
Cardiopulmonary bypass time (min)	100 (77, 129)	100 (77, 129)	100 (76, 130)	0.777
Aortic cross clamp time (min)	67 (47, 88)	67 (47, 88)	67 (47, 89)	0.917
Intraoperative transfusion of RBC (units)	1 (0, 3)	1 (0, 3)	1 (0, 3)	0.962

*RBC, red blood cell*.

### Development of the Full Nomogram Model

**Figure 2:**
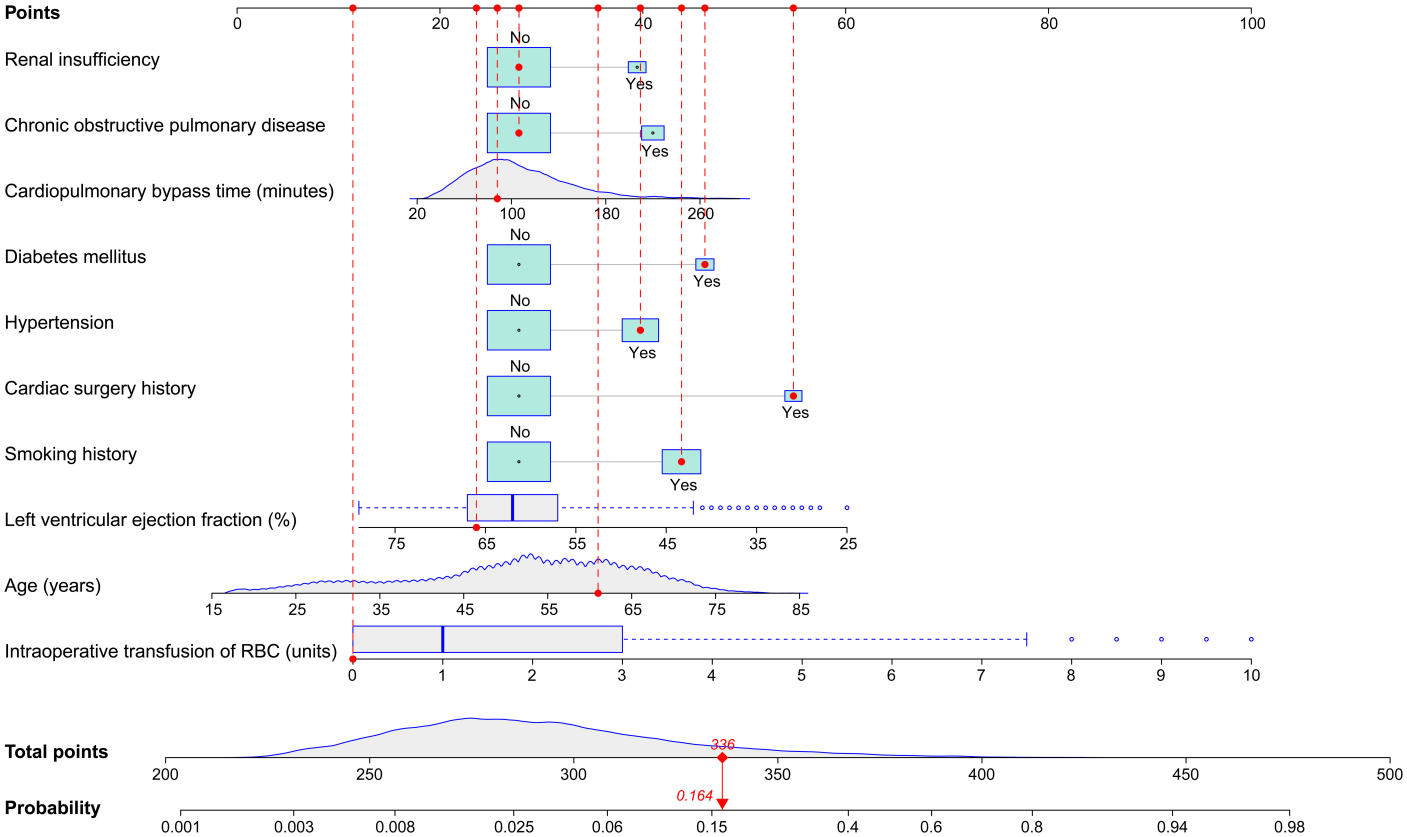
Nomogram for the prediction of POP after ECS. A specific patient was shown to illustrate how to use the nomogram. This was a 61-year-old patient who had a smoking history, hypertension, diabetes mellitus, previous cardiac surgery, a left ventricular ejection fraction of 66%, a cardiopulmonary bypass time of 88 min, without chronic obstructive pulmonary disease, renal insufficiency and RBC transfusion. The individual item score corresponding to each factor was presented at the top, and the total points were obtained from the sum of the scores corresponding to each factor by a red dot. Given values of the 10 predictors, the patient can be intuitively mapped onto the nomogram. It can be clearly seen from the nomogram that the total points of this patient was 336 points and the corresponding probability of POP was 0.164. ECS, elective cardiac surgery; POP, postoperative pneumonia; RBC, red blood cell.

In the training set, we conducted univariate analysis of potential predictors for POP after ECS and presented the results in Table 2. Factors with *P* < 0.1 were further analyzed by multivariate logistic regression and ten independent risk factors were identified, including advanced age, smoking history, diabetes mellitus, hypertension, renal insufficiency, COPD, lower left ventricular ejection fraction, cardiac surgery history, longer CPB time, and intraoperative RBC transfusion (Table 3). A full nomogram model was established to predict the probability of POP after ECS based on the full multivariate logistic regression model (Figure 2). Coefficients of the variables were scaled to scores within the range of 0 to 100, reflecting their relative importance. The individualized probability of POP after ECS can be directly and easily predicted by summing the corresponding scores. A specific case is presented in Figure 2.

**Table 2:** Univariate analysis of possible risk factors for POP after elective cardiac surgery in the training set.

**Characteristic**	**Without POP**	**With POP**	**χ^2^/Z/t**	***P*** **-value**
	***n*** **= 8,758 (%)**	***n*** **= 608 (%)**		
**Demographics**				
Age (years)	51.01 ± 13.15	59.60 ± 11.22	18.030	<0.001
Male	4,700 (53.7)	394 (64.8)	28.428	<0.001
Body mass index (kg/m^2^)	23.11 ± 3.28	23.54 ± 3.53	2.886	0.004
Smoking history	2,294 (26.2)	258 (42.4)	75.650	<0.001
Drinking history	1,742 (19.9)	146 (24.0)	6.004	0.014
**Underlying conditions**				
Hypertension	2,057 (23.5)	281 (46.2)	156.814	<0.001
Diabetes mellitus	596 (6.8)	118 (19.4)	128.225	<0.001
Chronic obstructive pulmonary disease	915 (10.4)	140 (23.0)	89.997	<0.001
Cerebrovascular disease	1,474 (16.8)	160 (26.3)	35.517	<0.001
Peripheral vascular disease	1,857 (21.2)	191 (31.4)	34.696	<0.001
Renal insufficiency	554 (6.3)	127 (20.9)	178.821	<0.001
Gastrointestinal tract disease	717 (8.2)	51 (8.4)	0.031	0.861
Atrial fibrillation	1,663 (19.0)	139 (22.9)	5.490	0.019
General surgery history	2,541 (29.0)	212 (34.9)	9.391	0.002
Cardiac surgery history	540 (6.2)	100 (16.4)	94.403	<0.001
New York Heart Association class III-IV	1,381 (15.8)	185 (30.4)	87.740	<0.001
Pulmonary artery hypertension	2,521 (28.8)	195 (32.1)	2.984	0.084
Pericardial effusion	1,098 (12.5)	105 (17.3)	11.375	0.001
Left ventricular ejection fraction (%)	62 (57, 67)	60 (55, 65)	11.102	<0.001
**Laboratory values**				
White blood cell count (× 10^9^/L)	5.65 (4.71, 6.80)	5.78 (4.80, 6.92)	0.775	0.083
Red blood cell count (× 10^12^/L)	4.31 (3.96, 4.67)	4.16 (3.76, 4.51)	0.574	<0.001
Hemoglobin (g/l)	130 (118, 141)	127 (114, 140)	0.260	<0.001
Serum creatinine (μmol/L)	70.8 (60.2, 83.3)	79.6 (65.3, 95.0)	6.781	<0.001
Serum albumin (g/L)	40.67 ± 3.78	39.43 ± 3.96	3.191	<0.001
Serum globulin (g/L)	24.61 ± 4.35	25.15 ± 4.84	2.691	0.007
**Operative variables**				
Cardiopulmonary bypass time (min)	98 (76, 127)	121 (95, 154)	19.953	<0.001
Aortic cross clamp time (min)	66 (47, 87)	78 (59, 101)	15.107	<0.001
Intraoperative transfusion of RBC (units)	1 (0, 2.5)	4 (1.5, 7)	17.825	<0.001

*POP, postoperative pneumonia; RBC, red blood cell*.

**Table 3:** Multivariate analysis of independent risk factors for POP after elective cardiac surgery.

**Characteristic**	**Coefficient**	**Standard error**	**OR (95% CI)**	***P*** **-value**
Smoking history	0.637	0.098	1.891 (1.562–2.290)	<0.001
Diabetes mellitus	0.729	0.127	2.073 (1.615–2.661)	<0.001
Chronic obstructive pulmonary disease	0.525	0.119	1.690 (1.339–2.134)	<0.001
Renal insufficiency	0.463	0.127	1.589 (1.240–2.038)	<0.001
Age (years)	0.033	0.005	1.033 (1.024–1.043)	<0.001
Left ventricular ejection fraction (%)	−0.035	0.005	0.965 (0.956–0.975)	<0.001
Hypertension	0.476	0.101	1.610 (1.321–1.962)	<0.001
Cardiopulmonary bypass time (min)	0.005	0.001	1.005 (1.003–1.007)	<0.001
Transfusion of RBC (units)	0.352	0.019	1.422 (1.369–1.477)	<0.001
Cardiac surgery history	1.076	0.141	2.932 (2.224–3.867)	<0.001
Intercept	−4.626	0.416	0.010	<0.001

*CI, confidence interval; OR, odds ratio; POP, postoperative pneumonia; RBC, red blood cell*.

### Validation and Assessment of the Full Nomogram Model

**Figure 3:**
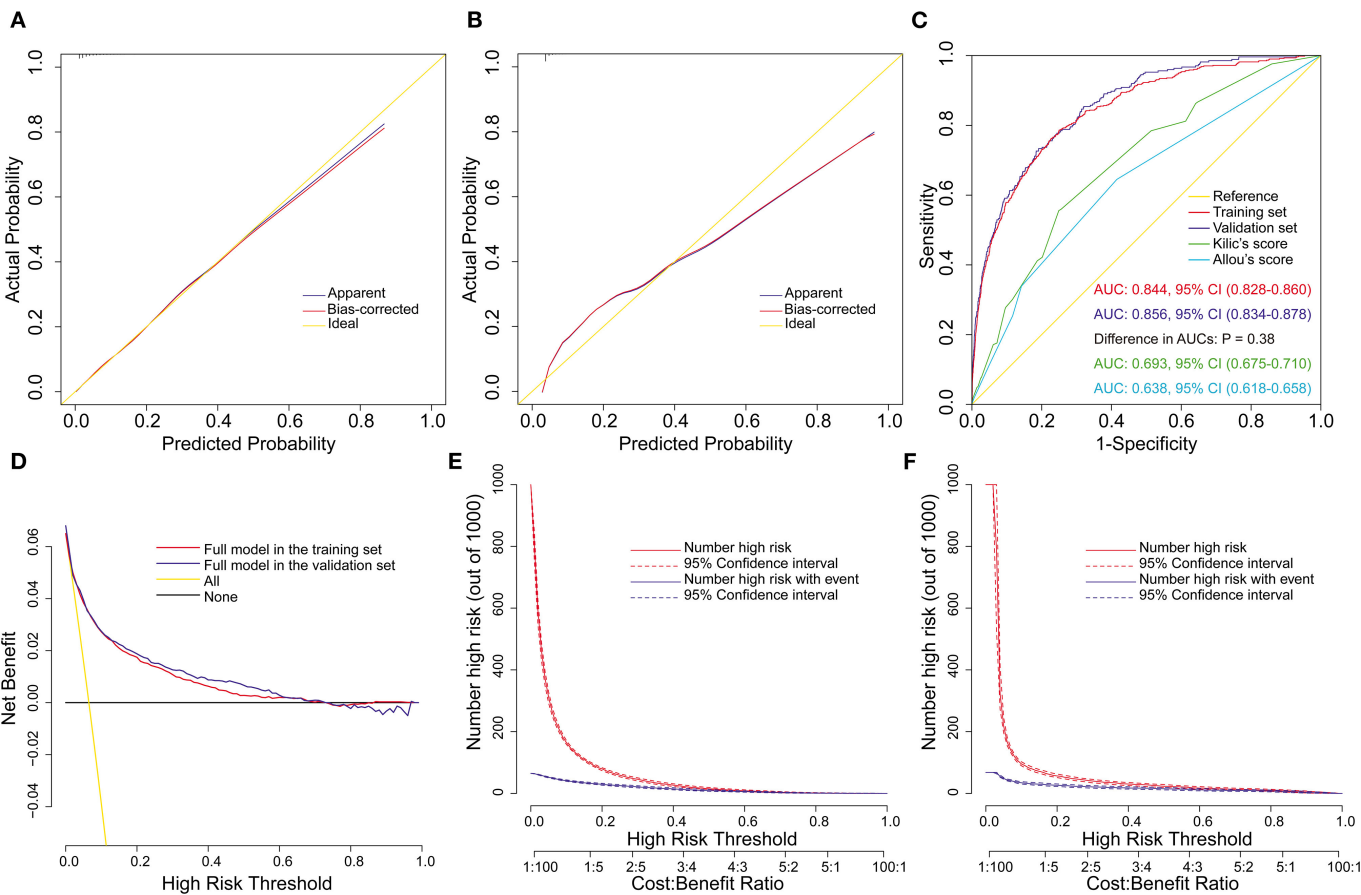
Assessment of the full nomogram model for POP after ECS. Calibration plots in the training set **(A)** and the validation set **(B)**, ROC curves in the two sets and comparison with previous models **(C)**, decision curves in the two sets **(D)**, and clinical impact curves in the training set **(E)** and the validation set **(F)**. AUC, area under the receiver operating characteristic curve; CI, confidence interval; ECS, elective cardiac surgery; POP, postoperative pneumonia; ROC, receiver operating characteristic curve.

The full nomogram was well validated by internal validation using 1,000 bootstrap replications and external validation in the validation set. By visual inspection of the calibration plots, the nomogram was well calibrated in both the training set (Hosmer-Lemeshow χ^2^ = 2.758, *P* = 0.949; Figure 3A) and the validation set (Hosmer-Lemeshow χ^2^ = 7.641, *P* = 0.469; Figure 3B). To assess the discriminative power of the nomogram, the ROC curves in the two sets were drawn (Figure 3C). The AUC was respectively, 0.844 (95% CI, 0.828–0.860) and 0.856 (95% CI, 0.834–0.878) in the two sets, indicating good discrimination, without significant difference (*P* = 0.38). Compared with published risk prediction models, the nomogram outperformed Allou's score (AUC: 0.638; 95% CI, 0.618–0.658) and Kilic's score (AUC: 0.693; 95% CI, 0.675–0.710) in predicting POP (*P* < 0.001; Figure 3C). The decision curves in the two sets indicated that the nomogram could obtain more clinical net benefits compared with “no intervention” and “intervention for all” strategies (Figure 3D). The clinical impact curves also demonstrated excellent predictive ability and showed good clinical usefulness (Figures 3E,F).

### Development, Validation, and Assessment of the Preoperative Nomogram Model

**Figure 4:**
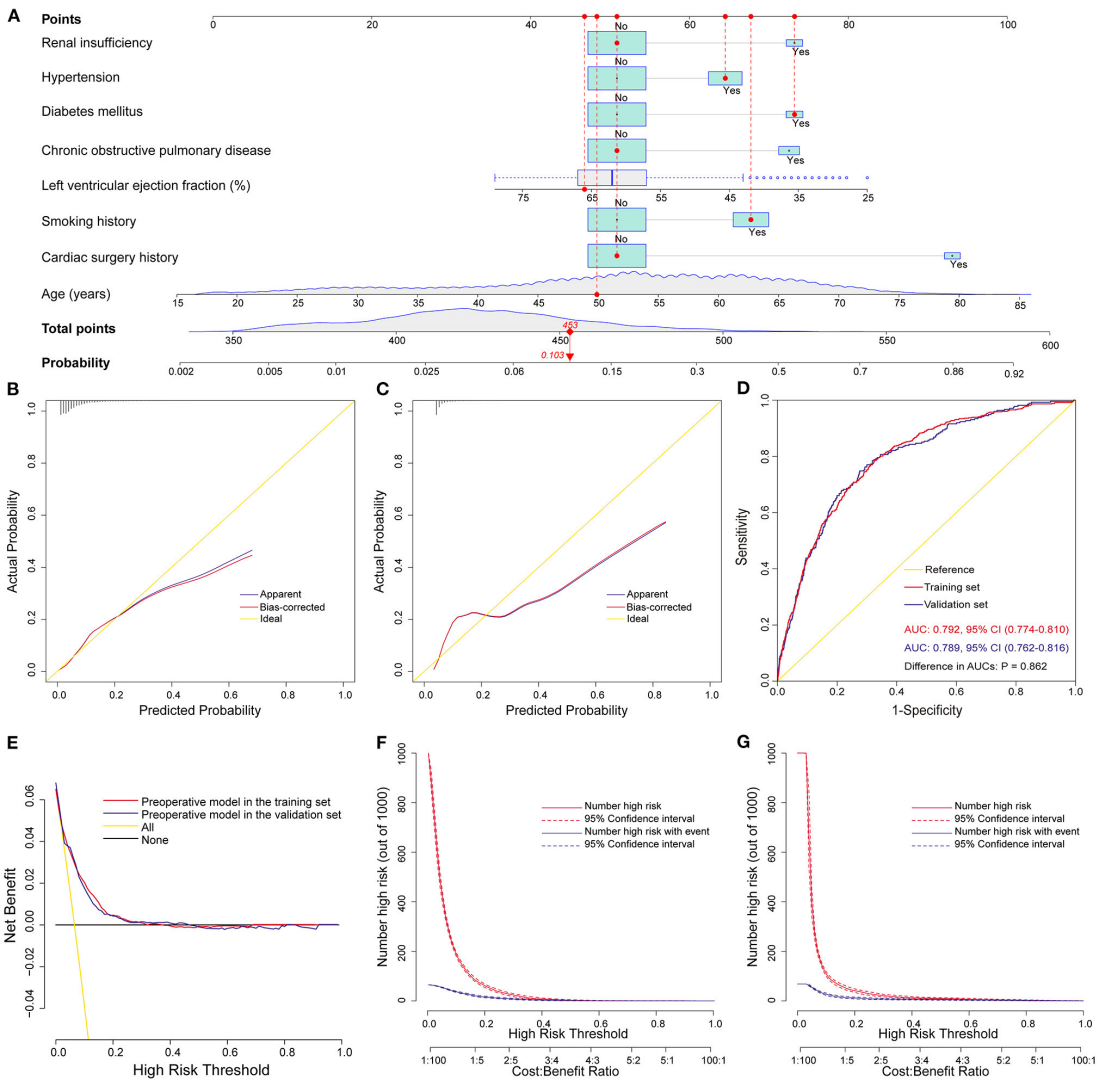
Development, validation, and assessment of the preoperative nomogram model for POP after ECS. Preoperative nomogram for POP after ECS **(A)**; A specific patient was shown to illustrate how to use the preoperative nomogram. This was a 50-year-old patient who had a smoking history, hypertension, diabetes mellitus, a left ventricular ejection fraction of 66%, without chronic obstructive pulmonary disease, renal insufficiency and previous cardiac surgery. The individual item score corresponding to each factor was presented at the top, and the total points were obtained from the sum of the scores corresponding to each factor by a red dot. Given values of the 8 predictors, the patient can be intuitively mapped onto the nomogram. It can be clearly seen from the nomogram that the total points of this patient was 453 points and the corresponding probability of POP was 0.103. Calibration plots in the training set **(B)** and the validation set **(C)**, ROC curves in the two sets **(D)**, decision curves in the two sets **(E)**, and clinical impact curves in the training set **(F)** and the validation set **(G)**. AUC, area under the receiver operating characteristic curve; CI, confidence interval; ECS, elective cardiac surgery; POP, postoperative pneumonia; ROC, receiver operating characteristic curve.

We constructed the above nomogram model using both pre- and intra-operative variables. For the convenience of clinical application, we further constructed a preoperative model, in which only factors available before surgery were included. Eight significant risk factors were identified by univarite and multivariate logistic regression analysis in the training set (Table 4), and then a preoperative nomogram was constructed (Figure 4A).

**Table 4:** Multivariate analysis of preoperative predictors for POP after elective cardiac surgery.

**Characteristic**	**Coefficient**	**Standard error**	**OR (95% CI)**	***P*** **-value**
Age (years)	0.051	0.004	1.053 (1.044–1.062)	<0.001
Smoking history	0.572	0.092	1.772 (1.481–2.121)	<0.001
Diabetes mellitus	0.759	0.121	2.136 (1.685–2.708)	<0.001
Chronic obstructive pulmonary disease	0.736	0.110	2.087 (1.682-2.589)	<0.001
Renal insufficiency	0.758	0.119	2.135 (1.692–2.694)	<0.001
Cardiac surgery history	1.433	0.131	4.191 (3.245–5.414)	<0.001
Hypertension	0.463	0.095	1.589 (1.318–1.916)	<0.001
Left ventricular ejection fraction (%)	−0.029	0.005	0.971 (0.962–0.980)	<0.001
Intercept	−4.579	0.384	0.010	<0.001

*CI, confidence interval; OR, odds ratio; POP, postoperative pneumonia*.

This model was also well validated by both internal validation and external validation. We assessed the goodness of fit of the nomogram by visual inspection of the calibration plots (Figures 4B,C) and the Hosmer-Lemeshow goodness-of-fit test. Both indicated good calibration, with Hosmer-Lemeshow χ^2^ values of 14.284 (*P* = 0.075) and 11.898 (*P* = 0.156) in the training and validation sets, respectively. The AUC was 0.792 (95% CI, 0.774–0.810) in the training set and 0.789 (95% CI, 0.762–0.816) in the validation set (Figure 4D), without significant difference (*P* = 0.86). The decision curve analysis demonstrated that the nomogram model had usefulness in clinical practice (Figures 4E–G).

### Risk Stratification

**Figure 5:**
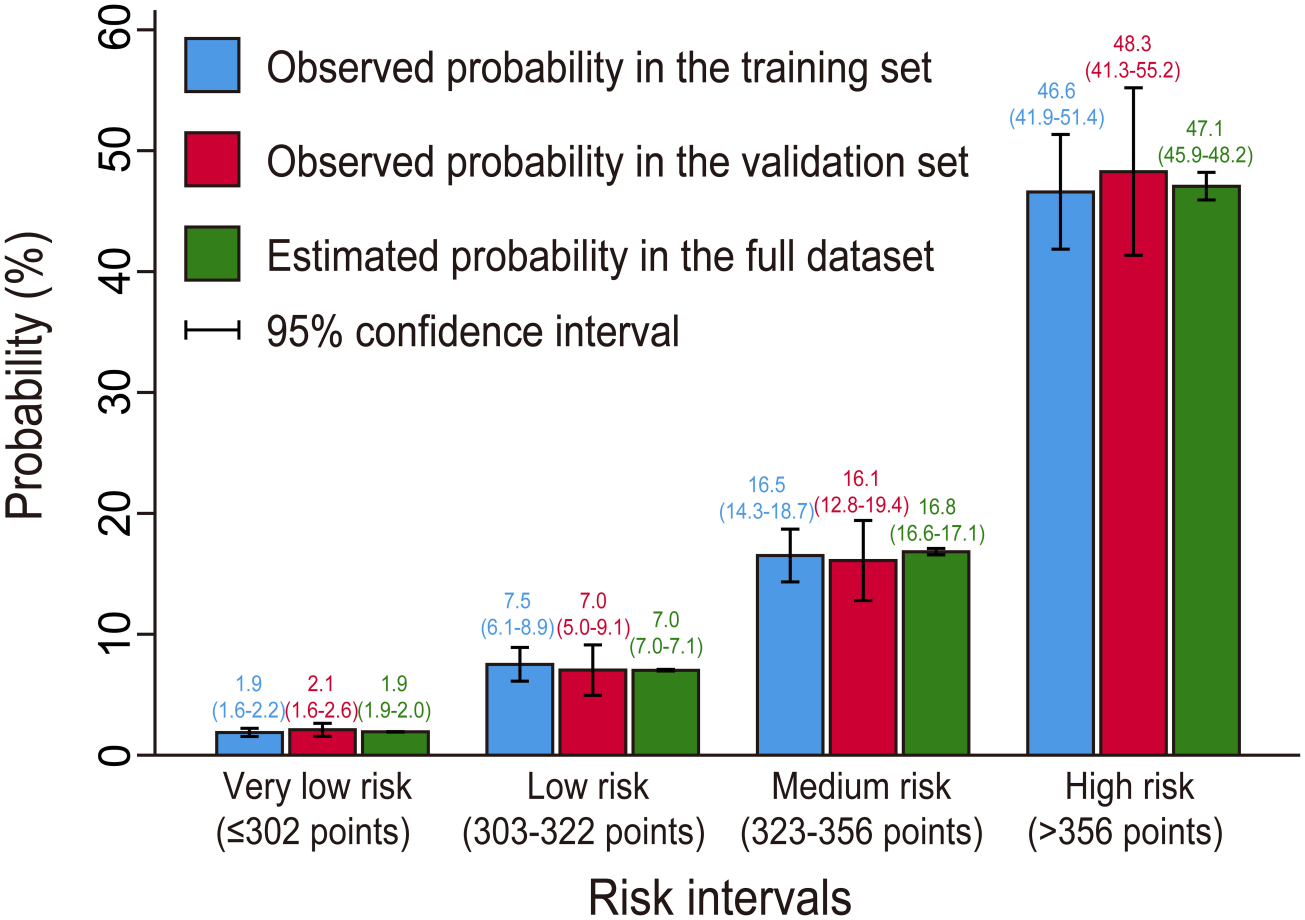
Bar chart showing the agreement between estimated and observed probabilities.

We further defined four risk intervals as very low, low, medium, and high risk groups for POP on the basis of the full nomogram model and clinical practice. The cutoff values were selected as 302, 322, and 356 points, corresponding to estimated probabilities of 0.05, 0.1, and 0.3 (Table 5). In this study, more than two-thirds of the patients were classified into the very low risk group, 14.6% into the low risk group, 11.9% into medium risk group, and only 4.7% into the high risk group. Estimated probabilities and observed probabilities in the two sets of the four risk intervals are presented in Figure 5, indicating good consistency.

**Table 5:** Risk intervals of POP based on the full nomogram model.

**Risk intervals**	**Very low risk**	**Low risk**	**Medium risk**	**High risk**
	**(≤302 points)**	**(303–322 points)**	**(323–356 points)**	**(>356 points)**
Estimated probability (%)	<5	5–10	10–30	>30
Observed probability, % (95% CI)	2.0 (1.7–2.2)	7.4 (6.2–8.5)	16.4 (14.6–18.2)	47.1 (43.2–51.1)
No. of patients (%)	9,206 (68.8)	1,952 (14.6)	1,592 (11.9)	630 (4.7)

*CI, confidence interval; POP, postoperative pneumonia*.

### Outcome

The overall mortality rate was 2.0%, but it increased significantly in patients with POP compared to patients without that (25.1 vs. 0.4%, *P* < 0.001). Similarly, we observed obviously higher risks of reintubation, tracheotomy, and readmission to ICU in patients with POP. In addition, the postoperative length of ICU and hospital stay were also significantly longer in these patients. Details are compared between patients with and without POP after ECS (Table 6).

**Table 6:** Postoperative variables in patients with and without POP after elective cardiac surgery.

**Outcome**	**All patients**	**Without POP**	**With POP**	***P*** **-value**
	***n*** **= 13,380 (%)**	***n*** **= 12,498 (%)**	***n*** **= 882 (%)**	
Reintubation	429 (3.2)	114 (0.9)	315 (35.7)	<0.001
Tracheotomy	168 (1.3)	18 (0.1)	150 (17.0)	<0.001
Readmission to ICU	413 (3.0)	192 (1.5)	221 (25.1)	<0.001
ICU stay (days)	3 (2, 4)	3 (2, 4)	10 (7, 16)	<0.001
Hospital stay (days)	14 (11, 18)	13 (10, 17)	25 (19, 33)	<0.001
Mortality	277 (2.0)	56 (0.4)	221 (25.1)	<0.001

*ICU, intensive care unit; POP, postoperative pneumonia*.

## Discussion

POP is now recognized as an important cause of morbidity and mortality in patients undergoing cardiac surgery (12), which was further confirmed by the present study. The observed rate of POP was 6.6% in this study, within the range reported in the literature (5). The overall mortality was 2.0%, similar to other reports (13, 14). The mortality and other poor outcomes increased significantly among patients with POP, consistent with the results in the literature (2, 15), emphasizing the importance of identifying predictors and high-risk patients.

Some studies focused on POP following cardiac surgery have been conducted and several predictive rules have been established (6–9), however, none of these models performed well when applied to the present study population. This may be because most of the studies were done in developed countries such as Europe and the United States, and there are discrepancies regarding to ethnic groups and clinical baseline characteristics compared with Asians. To the best of our knowledge, this study is the first large-sized multicenter study to develop and validate nomogram models for POP after ECS. In this study, we used data from 13,380 patients undergoing ECS to develop and validate two multivariate nomogram models for POP. Eight preoperative independent risk factors and two intraoperative independent risk factors were identified. A full and a preoperative nomogram model were then constructed based on these predictors. Finally, four risk intervals were defined as very low, low, medium and high risk groups.

Although independent predictors for POP vary greatly in different reports, several patient characteristics have been widely recognized (1). (8) first conducted a multicenter study and established a prediction model for POP after coronary artery bypass grafting identifying that smoking history, COPD and serum creatinine level >1.2 mg/dL were independently associated with the increased risk of POP (8). Another multicenter prospective study conducted by (6) found that 17 preoperative factors including older age, diabetes mellitus, smoking history, chronic lung disease and lower ejection fraction were independent predictors for POP following coronary artery bypass grafting (6). (7) developed and validated a simplified risk score for POP in patients undergoing cardiac surgery claiming that age ≥65 years, poor cardiac function and chronic lung disease were associated with increased risk of POP (7). Another multicenter prospective study conducted by Ailawadi obtained similar results (9).

Cardiac surgery history was identified as the most important preoperative risk factors in our analysis. In comparison with initial cardiac operations, redo cardiac surgery carries an inherent risk for adverse outcomes and relates to an increased risk of morbidity and mortality (16). Complications after redo cardiac surgery are more common owing to more complex anatomy and operation (17). A redo sternotomy is challenging when the aorta and the heart are closely adhered to the back of the sternum. A prospective study conducted by (18) indicated that patients with previous cardiac surgery had a 3-fold increased risk of POP following cardiac surgery (18). Another multicenter study conducted by Kinlin et al. reported that the odds of experiencing POP after coronary artery bypass grafting were 2.3 times higher in patients with prior internal mammary artery graft (8). Valentino et al. compared the redo and first-time cardiac operations through a large propensity-matched series finding that there were significantly more comorbidities in the redo cardiac surgery group (16). Blood product transfusion, prolonged ventilator requirements and operative mortality were also increased for the reoperative cohort. (19) revealed that compared with patients without cardiac surgery history, patients with cardiac surgery history had advanced age, more comorbidities, more concomitant procedures, more intraoperative blood transfusion, and longer CPB time (19).

Unsurprisingly, the length of CPB was independently related to the development of POP following ECS in our analysis, which was consistent with most of the existing reports (1). Olga et al. reported that the duration of CPB was an independent risk factor for the development of POP following cardiac surgery, with an increased risk of 1% per min (20). A meta-analysis conducted by He et al. indicated that prolonged CPB may significantly increase the probability of POP following cardiac surgery. (7) reported that patients who underwent CPB > 100 min had a 1.71-fold higher risk of POP following cardiac surgery (7). Positive association between the risk of POP and the CPB time was also reported by (21). In their multivariate analysis, the risk of POP increased with the duration of CPB. After inclusion and selection of interactions, the interaction between duration of CPB >60 min and intraoperative RBC transfusion was identified as an independent predictors in their final model, with a 2.98-fold increased risk of POP in those patients (21).

CPB can reduce pulmonary compliance and lead to pulmonary dysfunction by inducing ischemia reperfusion injuries and systemic inflammatory responses (22). However, the importance of CPB is often underestimated or even neglected by some cardiac surgeons. Recently, minimal invasive extracorporeal circulation has been introduced and reported to be able to markedly improve clinical outcomes (23). This system have initiated important efforts within science and technology to further improve the biocompatibility of CPB components to minimize the adverse effects (24). Current clinical evidence have justified the superiority of minimal invasive extracorporeal circulation over conventional CPB in reducing haemodilution and better preserving haematocrit, which may reduce the need for perioperative blood transfusion. Furthermore, this system can significantly attenuate systemic inflammatory response and preserve end-organ function (24).

Minimal invasive extracorporeal circulation is a closed system which allows optimal perfusion. The system can provide systemic vascular resistance close to normal values and higher mean arterial pressure for any given flow, which may reduce the need for vasoactive drugs (23). In the past decade, this system has been developed as the best available perfusion technology in clinical practice. A large meta-analysis by (25) indicated that the use of minimal invasive extracorporeal circulation can significantly reduce postoperative morbidity and mortality in cardiac surgery compared to conventional CPB (25). Recently, they have also introduced the concept of a “more physiologic” cardiac surgery to highlight the requirement for further improvement of patient's outcomes (26).

The other independent intraoperative predictor for POP after ECS identified in our analysis was the volume of RBC transfusion. Transfusions of blood and blood products during cardiac surgery is common and can be lifesaving, however, there is growing evidence that blood transfusion is associated with side effects (27, 28). As evidenced by the prospective multicenter study of (29) the risk of POP after cardiac surgery increased 3.4 times in patients receiving blood transfusion and there was a substantial increase with each unit increase of transfused RBC (29). Another multicenter study conducted by (30) demonstrated that blood product conservation was related to improved outcomes and reduced costs after cardiac surgery. There was a significant reduction in both the risk of adverse events, such as POP and death, and the health care costs when implementing restricted perioperative blood transfusion strategy (30).

The changes of immune function can partially explain the relationship between the development of POP and blood transfusion (31). There is also an opinion that the risk of POP is related to the length of blood storage. The capacity of carrying oxygen may reduce and the inflammatory reactions related to transfusion may expand with time, leading to poor outcomes (32, 33). Several clinical practice guidelines have been developed for the management of blood transfusion, in which a restrictive transfusion strategy is especially recommended (34, 35). Given the numerous latent risks of transfusion, studies focusing on exploring alternative therapies may be a novel direction.

Several reported predictors for POP following cardiac surgery in the literature were not identified as independent predictors in our analysis, such as cerebrovascular and peripheral vascular disease (6–8). This discrepancy may be caused by different demographic characteristics and comorbidities of the study populations. The duration of mechanical ventilation has been frequently reported to be associated with the development of POP, as well as reintubation and tracheotomy (9, 18, 36). This may be explained by the fact that endotracheal intubation and tracheotomy can significantly damage the respiratory system's defense mechanism (37). However, we did not include these factors into multivariate analysis as candidate variables due to their nature of postoperative variables and cannot be available early.

The nomogram models may have an immense role in risk assessment, risk stratification, as well as identifying high-risk patients. Some preventive measures have been introduced these years, such as oropharyngeal nursing (38), subglottic secretion drainage (39), and respiratory physiotherapy (40). However, it may be not appropriate to apply these measures to all patients without selection because some are time consuming, expensive and laborious. Instead, the implementation of appropriate interventions and preventive measures for high-risk populations identified by the nomogram models may result in significant clinical benefits. In addition, the nomogram models may contribute to a better doctor-patient communication in the medical setting, which is important and cannot be neglected.

### Limitations

This study has limitations due to its retrospective nature. First of all, although the standardized diagnostic criteria for POP had been established before study initiation, we cannot completely deny that some degree of variability and subjectivity may exist when clinically diagnosed. This may have allowed for over- or underestimation of the real incidence of POP. A well-designed prospective study in the future may be required to obtain a more accurate estimation of POP incidence. Second, some possible predictors that may affect the development of POP were not included in the multivariate analysis, such as drug use and surgery types. Even so, the nomograms have performed well in terms of calibration, discrimination, and clinical usefulness. Third, the clinical and outcome data were only documented during patient's hospitalization, which may lead to underestimation of morbidity and mortality. It would be of interest to perform long-term follow-up in subsequent work.

## Conclusions

To our knowledge, this is the first study reporting nomogram models for POP after ECS. Eight preoperative variables and two intraoperative variables were identified as independent risk factors by multivariate logistic regression analysis. A preoperative nomogram model and a full nomogram model were developed and well validated. Both nomograms performed well in terms of calibration and discrimination, and may have good clinical usefulness. Risk stratification was performed and four risk intervals were identified. The nomogram models may help improve clinical decision making through individualized risk estimation and high-risk patients identification.

## Data Availability Statement

The raw data supporting the conclusions of this article will be made available by the authors, without undue reservation.

## Ethics Statement

The studies involving human participants were reviewed and approved by Ethics Committee of Tongji Medical College of Huazhong University of Science and Technology (IORG No. IORG0003571). Written informed consent for participation was not required for this study in accordance with the national legislation and the institutional requirements.

## Author Contributions

XD, XH, AZ, and DW: conception and design. XD, HW, and SL: administrative support. XD, XH, and HW: provision of study materials or patients. DW, JW, FX, and XL: collection and assembly of data. DW, SL, AZ, and XH: data analysis and interpretation. All authors manuscript writing and final approval of manuscript.

## Funding

This work was supported by the National Natural Science Foundation of China (Grant Nos. 81800413 and 81801586).

## Conflict of Interest

The authors declare that the research was conducted in the absence of any commercial or financial relationships that could be construed as a potential conflict of interest.

## Publisher's Note

All claims expressed in this article are solely those of the authors and do not necessarily represent those of their affiliated organizations, or those of the publisher, the editors and the reviewers. Any product that may be evaluated in this article, or claim that may be made by its manufacturer, is not guaranteed or endorsed by the publisher.

## Abbreviations

AUC: area under the receiver operating characteristic curve
CI: confidence interval
COPD: chronic obstructive pulmonary disease
CPB: cardiopulmonary bypass
ECS: elective cardiac surgery
ICU: intensive care unit
POP: postoperative pneumonia
RBC: red blood cell
ROC: receiver operating characteristic curve.

## References

[1] He S Chen B Li W Yan J Chen L Wang X. Ventilator-associated pneumonia after cardiac surgery: a meta-analysis and systematic review. J Thorac Cardiovasc Surg. (2014) 148:3148–55. 10.1016/j.jtcvs.2014.07.107 25240522

[2] Tamayo E Alvarez FJ Martinez-Rafael B Bustamante J Bermejo-Martin JF Fierro I. Ventilator-associated pneumonia is an important risk factor for mortality after major cardiac surgery. J Crit Care. (2012) 27:18–25. 10.1016/j.jcrc.2011.03.008 21596516

[3] Greco G Shi W Michler RE Meltzer DO Ailawadi G Hohmann SF. Costs associated with health care-associated infections in cardiac surgery. J Am Coll Cardiol. (2015) 65:15–23. 10.1016/j.jacc.2014.09.079 25572505 PMC4293042

[4] Klompas M. Complications of mechanical ventilation–the CDC's new surveillance paradigm. N Engl J Med. (2013) 368:1472–5. 10.1056/nejmp1300633 23594002

[5] Fitch ZW Whitman GJ. Incidence, risk, and prevention of ventilator-associated pneumonia in adult cardiac surgical patients: a systematic review. J Card Surg. (2014) 29:196–203. 10.1111/jocs.12260 24304223

[6] Strobel RJ Liang Q Zhang M Wu X Rogers MA Theurer PF. A preoperative risk model for postoperative pneumonia after coronary artery bypass grafting. Ann Thorac Surg. (2016) 102:1213–9. 10.1016/j.athoracsur.2016.03.074 27261082 PMC5030150

[7] Kilic A Ohkuma R Grimm JC Magruder JT Sussman M Schneider EB. A novel score to estimate the risk of pneumonia after cardiac surgery. J Thorac Cardiovasc Surg. (2016) 151:1415–20. 10.1016/j.jtcvs.2015.12.049 27085620

[8] Kinlin LM Kirchner C Zhang H Daley J Fisman DN. Derivation and validation of a clinical prediction rule for nosocomial pneumonia after coronary artery bypass graft surgery. Clin Infect Dis. (2010) 50:493–501. 10.1086/649925 20085462

[9] Ailawadi G Chang HL O'gara PT O'sullivan K Woo YJ Derose JJ. Pneumonia after cardiac surgery: experience of the national institutes of health/canadian institutes of health research cardiothoracic surgical trials network. J Thorac Cardiovasc Surg. (2017) 153:1384–91. 10.1016/j.jtcvs.2016.12.055 28341473 PMC5439299

[10] Bonell A Azarrafiy R Huong V Viet TL Phu VD Dat VQ. A systematic review and meta-analysis of ventilator-associated pneumonia in adults in Asia: an analysis of national income level on incidence and etiology. Clin Infect Dis. (2019) 68:511–8. 10.1093/cid/ciy543 29982303 PMC6336913

[11] Kalil AC Metersky ML Klompas M Muscedere J Sweeney DA Palmer LB. Management of adults with hospital-acquired and ventilator-associated pneumonia: 2016 clinical practice guidelines by the infectious diseases society of America and the American thoracic society. Clin Infect Dis. (2016) 63:e61–e111. 10.1093/cid/ciw353 27418577 PMC4981759

[12] Torres A Niederman MS Chastre J Ewig S Fernandez-Vandellos P Hanberger H. International ERS/ESICM/ESCMID/ALAT guidelines for the management of hospital-acquired pneumonia and ventilator-associated pneumonia: guidelines for the management of hospital-acquired pneumonia (HAP)/ventilator-associated pneumonia (VAP) of the European respiratory society (ERS), European society of intensive care medicine (ESICM), European society of clinical microbiology and infectious diseases (ESCMID) and asociacion latinoamericana del torax (ALAT). Eur Respir J. (2017) 50:1700582. 10.1183/13993003.00582-2017 28890434

[13] Duchnowski P Hryniewiecki T Kusmierczyk M Szymanski P. Performance of the euroscore II and the society of thoracic surgeons score in patients undergoing aortic valve replacement for aortic stenosis. J Thorac Dis. (2019) 11:2076–81. 10.21037/jtd.2019.04.48 31285901 PMC6588761

[14] He S Wu F Wu X Xin M Ding S Wang J. Ventilator-associated events after cardiac surgery: evidence from 1,709 patients. J Thorac Dis. (2018) 10:776–83. 10.21037/jtd.2018.01.49 29607148 PMC5864661

[15] Wang D Huang X Wang H Le S Yang H Wang F. Risk factors for postoperative pneumonia after cardiac surgery: a prediction model. J thorac dis. (2021) 13:2351–62. 10.21037/jtd-20-3586 34012584 PMC8107540

[16] Bianco V Kilic A Gleason TG Aranda-Michel E Habertheuer A Wang Y. Reoperative cardiac surgery is a risk factor for long-term mortality. Ann Thorac Surg. (2020) 110:1235–42. 10.1016/j.athoracsur.2020.02.028 32199823

[17] Roselli EE Pettersson GB Blackstone EH Brizzio ME Houghtaling PL Hauck R. Adverse events during reoperative cardiac surgery: frequency, characterization, and rescue. J Thorac Cardiovasc Surg. (2008) 135:316–23. 10.1016/j.jtcvs.2007.08.060 18242260

[18] Hortal J Giannella M Perez MJ Barrio JM Desco M Bouza E. Incidence and risk factors for ventilator-associated pneumonia after major heart surgery. Intensive Care Med. (2009) 35:1518–25. 10.1007/s00134-009-1523-3 19557389

[19] Norton EL Rosati CM Kim KM Wu X Patel HJ Deeb GM. Is previous cardiac surgery a risk factor for open repair of acute type A aortic dissection? J Thorac Cardiovasc Surg. (2020) 160:8–17. 10.1016/j.jtcvs.2019.07.093 31585754 PMC7043015

[20] de la Varga-Martínez O Gómez-Sánchez E Muñoz MF Lorenzo M Gómez-Pesquera E Poves-Álvarez R. Impact of nosocomial infections on patient mortality following cardiac surgery. J Clin Anesth. (2021) 69:110104. 10.1016/j.jclinane.2020.110104 33221707

[21] Allou N Bronchard R Guglielminotti J Dilly MP Provenchere S Lucet JC. Risk factors for postoperative pneumonia after cardiac surgery and development of a preoperative risk score^*^. Crit Care Med. (2014) 42:1150–6. 10.1097/ccm.0000000000000143 24351376

[22] Mcdonald CI Fraser JF Coombes JS Fung YL. Oxidative stress during extracorporeal circulation. Eur J Cardio-Thorac. (2014) 46:937–43. 10.1093/ejcts/ezt637 24482384

[23] Anastasiadis K Argiriadou H Deliopoulos A Antonitsis P. Minimal invasive extracorporeal circulation (MIECC): the state-of-the-art in perfusion. J Thorac Dis. (2019) 11:s1507–14. 10.21037/jtd.2019.01.66 31293801 PMC6586580

[24] Anastasiadis K Murkin J Antonitsis P Bauer A Ranucci M Gygax E. Use of minimal invasive extracorporeal circulation in cardiac surgery: principles, definitions and potential benefits. a position paper from the minimal invasive extra-corporeal technologies international society (MiECTiS). Interact Cardiovasc Thorac Surg. (2016) 22:647–62. 10.1093/icvts/ivv380 26819269 PMC4892134

[25] Anastasiadis K Antonitsis P Haidich A Argiriadou H Deliopoulos A Papakonstantinou C. Use of minimal extracorporeal circulation improves outcome after heart surgery; a systematic review and meta-analysis of randomized controlled trials. Int J Cardiol. (2013) 164:158–69. 10.1016/j.ijcard.2012.01.020 22325958

[26] Anastasiadis K Antonitsis P Deliopoulos A Argiriadou H. A multidisciplinary perioperative strategy for attaining “more physiologic” cardiac surgery. Perfusion. (2017) 32:446–53. 10.1177/0267659117700488 28692337

[27] Crawford TC Magruder JT Fraser C Suarez-Pierre A Alejo D Bobbitt J. Less is more: results of a statewide analysis of the impact of blood transfusion on coronary artery bypass grafting outcomes. Ann Thorac Surg. (2018) 105:129–36. 10.1016/j.athoracsur.2017.06.062 29074154

[28] Sultan I Bianco V Aranda-Michel E Kilic A Serna-Gallegos D Navid F. The use of blood and blood products in aortic surgery is associated with adverse outcomes. J Thorac Cardiovasc Surg. (2021). 10.1016/j.jtcvs.2021.02.096. [Epub ahead of print]. 33838909

[29] Likosky DS Paone G Zhang M Rogers MA Harrington SD Theurer PF. Red blood cell transfusions impact pneumonia rates after coronary artery bypass grafting. Ann Thorac Surg. (2015) 100:794–800. 10.1016/j.athoracsur.2015.03.089 26209489 PMC4904229

[30] Lapar DJ Crosby IK Ailawadi G Ad N Choi E Spiess BD. Blood product conservation is associated with improved outcomes and reduced costs after cardiac surgery. J Thorac Cardiovasc Surg. (2013) 145:796–803. 10.1016/j.jtcvs.2012.12.041 23414992

[31] Karsten E Herbert BR. The emerging role of red blood cells in cytokine signalling and modulating immune cells. Blood Rev. (2020) 41:100644. 10.1016/j.blre.2019.100644 31812320

[32] Roback JD Neuman RB Quyyumi A Sutliff R. Insufficient nitric oxide bioavailability: a hypothesis to explain adverse effects of red blood cell transfusion. Transfusion. (2011) 51:859–66. 10.1111/j.1537-2995.2011.03094.x 21496047 PMC4793902

[33] Hod EA Brittenham GM Billote GB Francis RO Ginzburg YZ Hendrickson JE. Transfusion of human volunteers with older, stored red blood cells produces extravascular hemolysis and circulating non-transferrin-bound iron. Blood. (2011) 118:6675–82. 10.1182/blood-2011-08-371849 22021369 PMC3242722

[34] Carson JL Guyatt G Heddle NM Grossman BJ Cohn CS Fung MK. Clinical practice guidelines from the AABB: red blood cell transfusion thresholds and storage. JAMA. (2016) 316:2025–35. 10.1001/jama.2016.9185 27732721

[35] Jeffrey L Apfelbaum MD Gregory A Nuttall MD Richard T Connis PD. Practice guidelines for perioperative blood management: an updated report by the American society of anesthesiologists task force on perioperative blood management^*^. Anesthesiology. (2015) 122:241–75. 10.1097/aln.0000000000000463 25545654

[36] Hortal J Munoz P Cuerpo G Litvan H Rosseel PM Bouza E. Ventilator-associated pneumonia in patients undergoing major heart surgery: an incidence study in Europe. Crit Care. (2009) 13:r80. 10.1186/cc7896 19463176 PMC2717444

[37] Tobin M Manthous C. Mechanical ventilation. Am J Respir Crit Care Med. (2017) 196:p3–4. 10.1164/rccm.1962p3 28707967

[38] Bardia A Blitz D Dai F Hersey D Jinadasa S Tickoo M. Preoperative chlorhexidine mouthwash to reduce pneumonia after cardiac surgery: a systematic review and meta-analysis. J Thorac Cardiovasc Surg. (2019) 158:1094–100. 10.1016/j.jtcvs.2019.01.014 30826096

[39] Pozuelo-Carrascosa DP Herraiz-Adillo A Alvarez-Bueno C Anon JM Martinez-Vizcaino V Cavero-Redondo I. Subglottic secretion drainage for preventing ventilator-associated pneumonia: an overview of systematic reviews and an updated meta-analysis. Eur Respir Rev. (2020) 29:190107. 10.1183/16000617.0107-2019 32051169 PMC9488747

[40] Katsura M Kuriyama A Takeshima T Fukuhara S Furukawa TA. Preoperative inspiratory muscle training for postoperative pulmonary complications in adults undergoing cardiac and major abdominal surgery. Cochrane Database Syst Rev. (2015) CD010356. 10.1002/14651858.cd010356.pub2 26436600 PMC9251477

