# Supplementary material for: Development and Validation of Nomogram Models for Postoperative Pneumonia in Adult Patients Undergoing Elective Cardiac Surgery
Source: Front Cardiovasc Med. 2021 Oct 11;8:750828. doi: 10.3389/fcvm.2021.750828 (PMC8542719; doi:10.3389/fcvm.2021.750828)
Supplement: Supplementary file 1 [file Table_1.DOCX]

**The generation of the nomogram and related validation plots**

**First, copy the data set. Then, run the following programs.**

mydata<-read.table("clipboard",header = TRUE,sep = "\t")

mydata<-as.data.frame(mydata)

mydata$POP<-ifelse(mydata$POP=="1",1,0)

View(mydata)

head(mydata)

names(mydata)

summary(mydata)

str(mydata)

library(readr)

library(rms)

library(regplot)

attach(mydata)

dd<-datadist(mydata)

options(datadist='dd')

fit<-lrm(POP~.,data=mydata)

fit

nom<-regplot(fit,observation=mydata[1,],center=T,title="Nomogram",points=T, odds=F, showP=T, rank="sd", clickable=T)

cal<-calibrate(fit,method = 'boot',B=1000)

plot(cal,xlim = c(0,1.0),ylim = c(0,1.0))
